# Supplementary material for: Nanoencapsulation Boosts the Copper-Induced Defense Responses of a Susceptible Coffea arabica Cultivar against Hemileia vastatrix
Source: Antibiotics (Basel). 2023 Jan 26;12(2):249. doi: 10.3390/antibiotics12020249 (PMC9952095; doi:10.3390/antibiotics12020249)
Supplement: Supplementary file 1 [file antibiotics-12-00249-s001.zip › antibiotics-2159546-supplementary.pdf]

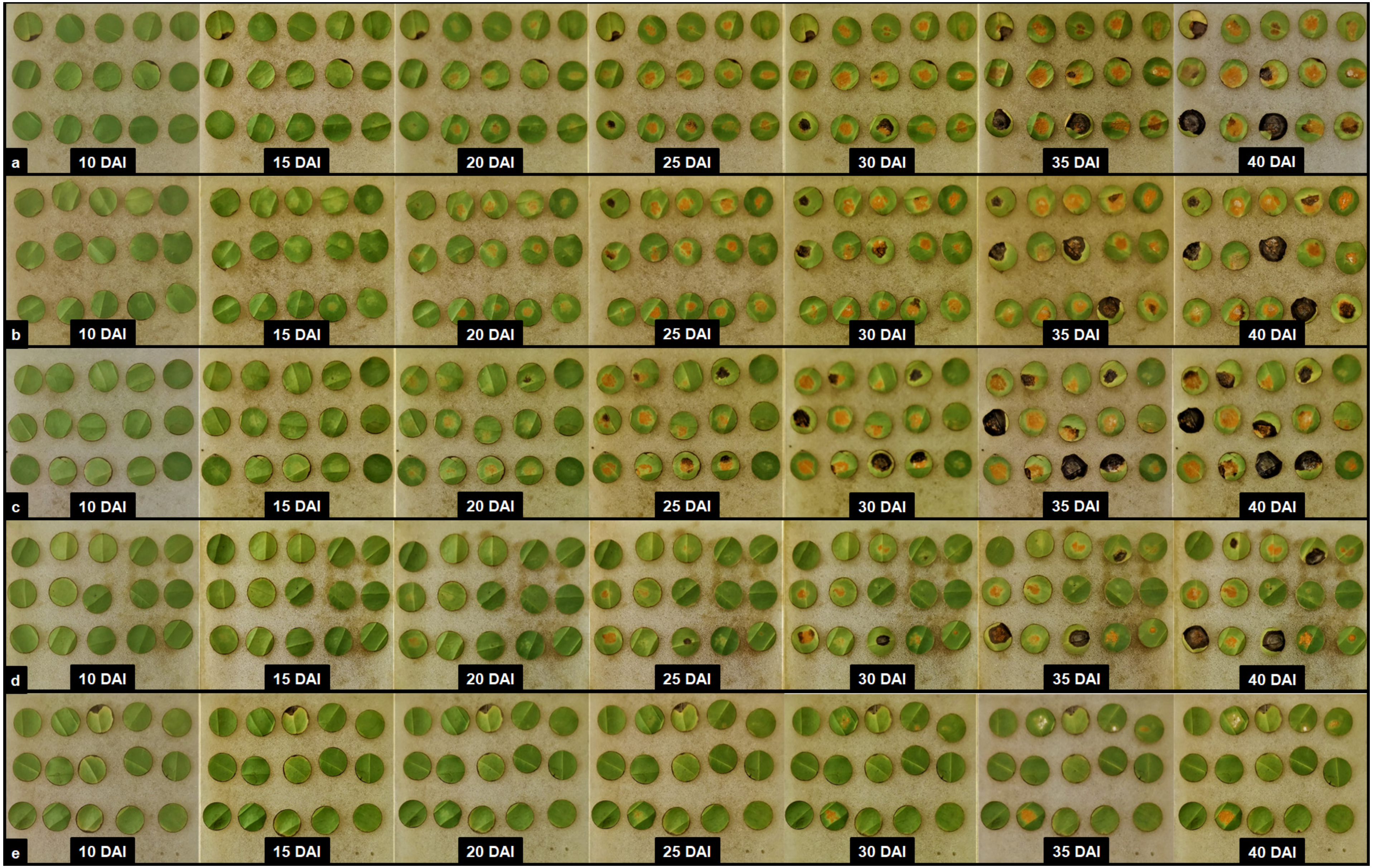

Figure S1. Representative images of *Hemileia vastatrix* disease progression in *Coffea arabica* cv. IPR 100 leaf discs at 10, 15, 20, 25, 30, 35, and 40 days after infection (DAI). The discs were treated with distilled water as a control (CONT) **(a)**, chitosan/sodium tripolyphosphate nanoparticles without Cu^{2+} ions (NP 0.25 g L^{-1}) **(b)**, (NP 0.5 g L^{-1}) **(c)**, (NP 1 g L^{-1}) **(d)**, and chitosan/sodium tripolyphosphate nanoparticles containing Cu^{2+} ions (NPCu 1.25 mmol L^{-1}) **(e)**.

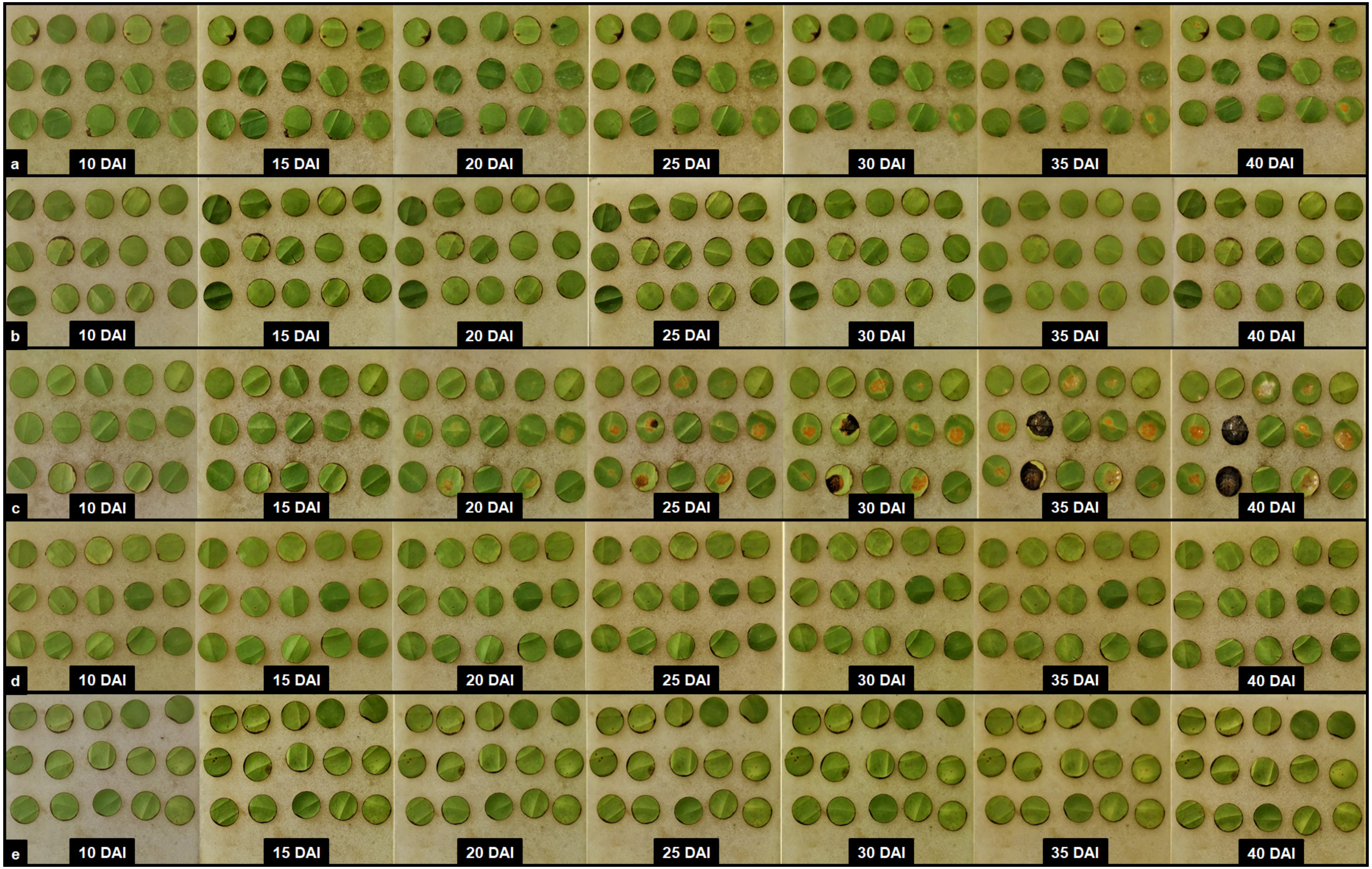

Figure S2. Representative images of *Hemileia vastatrix* disease progression in *Coffea arabica* cv. IPR 100 leaf discs at 10, 15, 20, 25, 30, 35, and 40 days after infection (DAI). The discs were treated with chitosan/sodium tripolyphosphate nanoparticles containing Cu^{2+} ions (NPCu 2.5 mmol L^{-1}) **(a)**, (NPCu 5 mmol L^{-1}) **(b)**, Cu^{2+} ions (Cu 1.25 mmol L^{-1}) **(c)**, (Cu 2.5 mmol L^{-1}) **(d),** and (Cu 5 mmol L^{-1}) **(e)**.

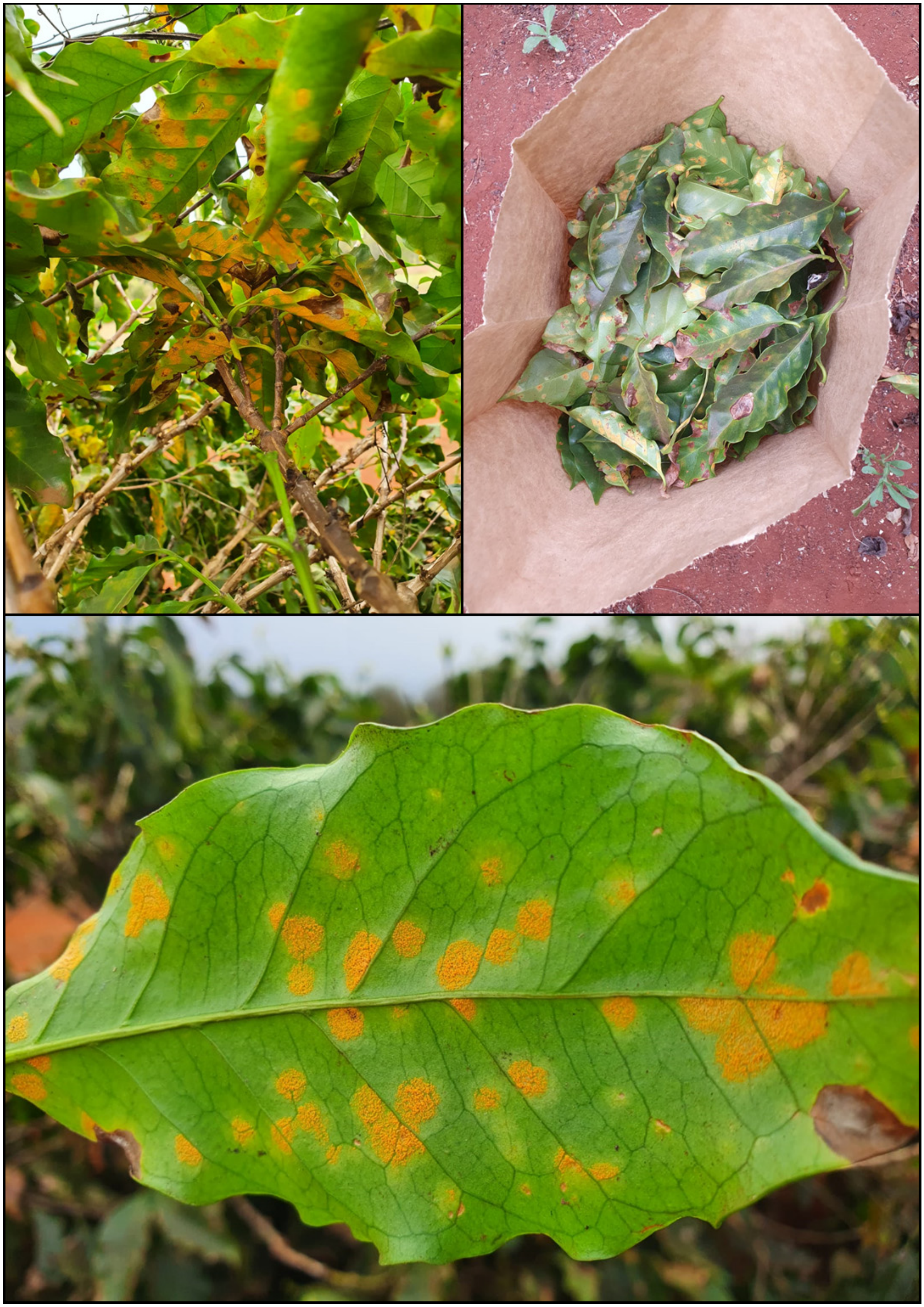

Figure S3. Field collection of coffee leaves containing urediniospores of *Hemileia vastatrix*.

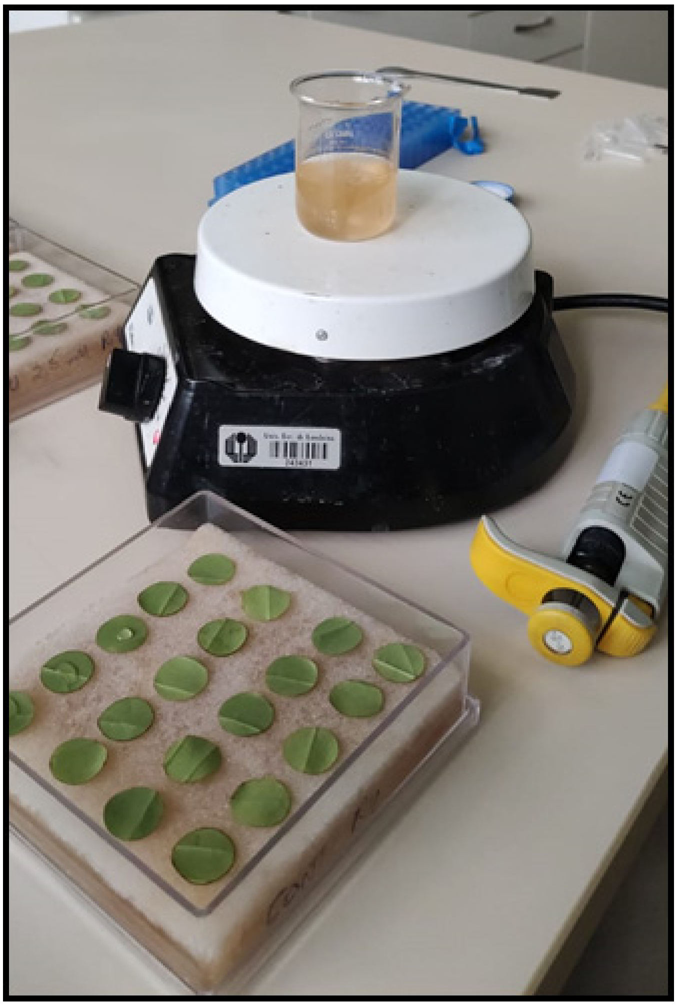

Figure S4. Suspension of viable *Hemileia vastatrix* urediniospores diluted in distilled water (1 mg mL^{-1}) and application of the inoculum on the abaxial face of *Coffea arabica* cv. IPR 100 leaf discs.
